# Author Correction: A small molecule stabilizer rescues the surface expression of nearly all missense variants in a GPCR

**DOI:** 10.1038/s41594-025-01734-y

**Published:** 2025-12-07

**Authors:** Taylor L. Mighell, Ben Lehner

**Affiliations:** 1 Centre for Genomic Regulation (CRG), The Barcelona Institute of Science and Technology, Barcelona, Spain; 2 Universitat Pompeu Fabra (UPF), Barcelona, Spain; 3 ICREA, Barcelona, Spain; 4 Wellcome Sanger Institute, Wellcome Genome Campus, Hinxton, United Kingdom

**Keywords:** Drug discovery, Genetics, Computational biology and bioinformatics

Correction to: *Nature Structural & Molecular Biology* 10.1038/s41594-025-01659-6, published online 23 September 2025.

In the version of this article initially published, in Fig. 4b, the representative structure shown for Tolvaptan was incorrect; the figure is now amended in the HTML and PDF versions of the article.

**Fig. 1:**
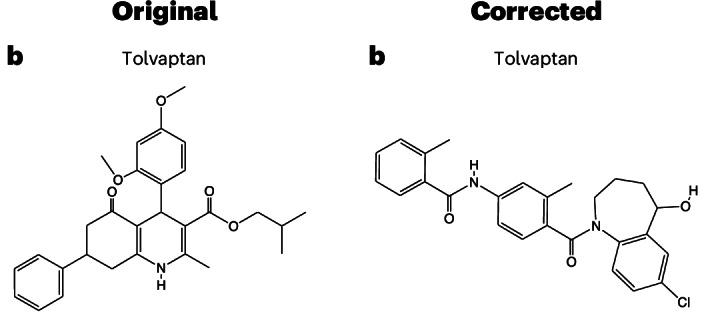
**Original and corrected Fig. 4b**.

